# Game-based self-regulated language learning: Theoretical analysis and bibliometrics

**DOI:** 10.1371/journal.pone.0243827

**Published:** 2020-12-16

**Authors:** Ruofei Zhang, Gary Cheng, Xieling Chen

**Affiliations:** 1 Department of English Language Education, The Education University of Hong Kong, Hong Kong SAR, China; 2 Department of Mathematics and Information Technology, The Education University of Hong Kong, Hong Kong SAR, China; Lingnan University, HONG KONG

## Abstract

Game-based learning and self-regulated learning have long been valued as effective approaches to language education. However, little research has been conducted to investigate their integration, namely, game-based self-regulated language learning (GBSRLL). This study aims to conceptualise GBSRLL based on the combination of theoretical analysis, thematic evolution analysis, and social network analysis on the research articles in the fields of game-based language learning and self-regulated language learning. The results show that GBSRLL is a new interdisciplinary field emerging since the period from 2018 to 2019. Self-regulated learning strategies that can be performed in GBSRLL, the effects of GBSRLL on learners’ affective states, and the features in GBSRLL were the prominent research topics in this field. Its theoretical foundation centres on the positive correlations between learner motivation, self-efficacy, and autonomy and the implementation of game-based learning and self-regulated learning. It is feasible to conduct GBSRLL due to the strong supportiveness of game mechanics for various phases and strategies of self-regulated learning. More contributions to this new interdisciplinary field are called for, especially from the aspects of the long-term effects of GBSRLL on academic performance and the useful tools and technologies for implementing GBSRLL.

## 1. Introduction

Having long been a focus of interest in the field of educational psychology [1], self-regulated learning has been increasingly valued as an effective approach to language education [2, 3]. In self-regulated language learning (hereinafter, SRLL), learners take charge of their learning actions, factors, and processes through various strategies [1, 4] and achieve positive learning perceptions and satisfying learning outcomes [5, 6]. Along with the rapid development of technology-enhanced language learning in recent years [7], this approach has been increasingly integrated with various types of modern technology [3], such as mobile tools [5], e-portfolios [8], online editors, and wikis [9].

Compared to the aforementioned types of technology, little research has been conducted on the integration of game mechanics into SRLL, that is game-based self-regulated language learning (hereinafter, GBSRLL). As an interdisciplinary field between game-based language learning (hereinafter, GBLL) and SRLL, however, GBSRLL may be highly potential for future research on language education, considering the positive correlations of GBLL and SRLL with learners’ motivation and self-efficacy at the theoretical level [10–13] and the probable supportiveness of game mechanics [14] for various phases and strategies of self-regulated learning at the practical level [15, 16]. Recently, increasingly more researchers have demonstrated their interest in GBSRLL and reported its effectiveness (e.g., [5, 12]). Hence, to call for more contributions to GBSRLL and provide implications for future researchers in this interdisciplinary field, it would be beneficial to throw light on this field by tracing its history, conceptualise it, and suggest possible directions for future expansion.

To address this issue, this study aims to analyse the research articles on GBLL and SRLL published from 2015 to 2020 through a mixed-methods approach. Four research questions were proposed to guide this study:

RQ1: What are the concepts of GBLL and SRLL?
RQ2: What is the history of the interdisciplinary field between GBLL and SRLL?
RQ3: What are the prominent research topics in the interdisciplinary field between GBLL and SRLL?
RQ4: What are the concepts and future directions of GBSRLL?

## 2. Materials and methods

This study was conducted through a four-step method, including: (1) creation of datasets, in which two sets of research articles were determined for analysis, corresponding to the topics of GBLL and SRLL; (2) theoretical analysis, in which the two datasets were analysed from the theoretical aspect, addressing RQ1; (3) thematic evolution analysis, in which the emergence of interdisciplinary topics was traced respectively in the fields of SRLL and GBLL, addressing RQ2; and (4) social network analysis, in which the relations between the academic research in the fields of GBLL and SRLL were explored and visualised, addressing RQ3. Based on the analytical results, RQ4 was then addressed.

### 2.1 Creation of datasets

The authors created two datasets for analysis corresponding to the fields of GBLL and SRLL. The data were retrieved from the Science Citation Index Extended (SCIE) and Social Science Citation Index (SSCI) in Web of Science (www.webofknowledge.com), with “2015-present” for the time span, “English” as the language, and “article” for the required document type.

The authors used keyword queries to retrieve the research articles that contained specific keywords in titles, abstracts, or author-defined keywords. The keyword queries were based on three groups of keywords. (a) For game-based learning, the keywords were provided by domain experts, specifically, “game,” “game-based,” “gamified,” and “gamification,” with the OR operators between them. (b) For self-regulated learning, the keywords were selected from the search terms used in two well-cited review studies in the field of SRLL [2] and [4], specifically, “self-regulated,” “self-regulation,” “self-regulatory,” “metacognitive strategy,” “motivational strategy,” “cognitive strategy,” “behavioural strategy,” “self-directed,” “self-access,” “self-controlled,” “self-guided,” “self-instructed,” “independent learning,” “autonomous learning,” and “autonomy,” with the OR operators between them. (c) For language learning, this study adopted the keywords used in a review of technology-enhanced language learning [7], specifically, “language learning,” “learn language,” “language teaching,” “teach language,” “language education,” and “language acquisition,” with the OR operators between them. The final keyword query for GBLL consisted of keyword groups (a) and (c), with the AND operators between them; the final keyword query for SRLL consisted of keyword groups (b) and (c), with the AND operators between them.

For quality assurance, the data retrieval was conducted in SCIE and SSCI databases, following previous review studies (e.g., [17, 18]), because of the wide recognition of them [19, 20]. At the prior stage of this study, we included book chapters, conference papers, non-SCIE and non-SSCI journal articles in the databases; after having read a few, however, we found problems of these papers and decided to exclude them. The research articles for analysis should present detailed explanations of the theoretical framework, explicit descriptions of the research method, and in-depth analyses of the research findings. Thus, the databases were finalised to be the publications of SCIE and SSCI journals.

The time span for data retrieval was from 2015 to the present (November 16^th^, 2020) because GBSRLL remains a very new field in which most research articles were published within the recent five years. Besides, only when the research articles published within the recent three to five years are analysed, the newest findings and latest trends of the target field can be attained due to the rapid development of methods and technologies of language education [7].

This step ended with 54 papers in the database on GBLL and 314 papers in the database on SRLL.

### 2.2 Theoretical analysis

To conceptualise GBLL and SRLL, the two datasets were analysed from four aspects, namely, (a) definitions, (b) theoretical frameworks, (c) main methods of implementation, and (d) effectiveness on language learning. The analyses were conducted based on the information as reported by the articles literally. The authors first analysed 10 studies together, five on GBLL and five on SRLL, to reach an agreement on the coding scheme. Subsequently, the authors analysed the remaining articles independently. The results of analyses were compared. The differences between the results were resolved via discussion and consultation with a fourth researcher who was an expert in the field. Our coding was finalised with satisfactory inter-rater reliability (Pearson’s *r* = 0.92).

### 2.3 Thematic evolution analysis

To trace the history of the interdisciplinary field, we explored the evolution of GBLL- or SRLL-related topics in the two databases to identify the emergence of the topics of GBLL in the field of SRLL and the emergence of the topics of SRLL in the field of GBLL. Specifically, in the domain of GBLL, we indicate the timeline detailing the time of publication of the GBLL papers by the X-axis and place each topic related to self-regulated learning and game-based learning in the corresponding timing averaged by the publication years of all the GBLL papers containing the topic. Topics are represented by nodes of different colours and sizes proportional to their frequencies in the corresponding dataset (i.e., on GBLL). A similar procedure was conducted for the domain of SRLL.

### 2.4 Social network analysis

Based on networks and graph theory, social network analysis was used to understand complex relations and interactions between social actors within a specific social context [21, 22]. In the analysis, the social actors were represented by nodes, and the relations and interactions were represented by the links between nodes [21]. Over the years, social network analysis has widely been applied in various disciplines of social science [23, 24], demonstrating a plentiful of benefits in discerning the roles and situations of different social actors, identifying the correlations between social actors, and presenting a comprehensive picture of the target context [25].

This study applied social network analysis to investigate the interdisciplinary field between the GBLL and SRLL in the context of language learning. It was investigated from two aspects: (a) the correlation of the SRLL studies with the GBLL-related topics, and (b) the correlation of the GBLL studies with the SRLL-related topics. The topics here are indicated by key terms extracted from the title and abstract of each article using a self-developed program based on Natural Language Processing Toolkit. We considered only terms related to GBLL or SRLL. The analysis was conducted using *Gephi* [26]. In the generated outputs, topics are represented by nodes of different colours and sizes proportional to their frequencies in the corresponding dataset. The links between the nodes indicate the topical correlations, the width of a link indicates the correlation strength as measured by the number of articles in which the linked topics co-occur. In other words, the thicker a link is between topics, the more likely the linked topics are discussed within a paper.

## 3. Results

This section reports the results of theoretical analysis, thematic evolution analysis, and social network analysis on the two datasets to address RQs 1, 2, and 3 subsequently.

### 3.1 Concepts of GBLL

Game-based learning is generally defined as learning through game playing [27, 28]. In the context of language learning, it refers to an educational approach in which language learning instructions and activities are situated into and reinforced by rich game mechanics [14, 28]. This study identifies five game mechanics that were frequently applied in GBLL, specifically, (a) Goals/Rules, that is the lucid explanations of the goals and ways of learning/playing [27, 28]; (b) Mystery/Fantasy, that is the interesting storyline and engaging scenarios of learning/playing [29]; (c) Interactivity/Feedback, that is the communications with other learners/players or computerised non-player characters (i.e., NPCs) in learning/playing [30]; (d) Competitions/Contests, that is the learning/playing activities in which learners/players try to perform better than their peers [31]; and (e) Rewards/Points, that is learners’/player’s winning of rewards or points because of their completion of or good performance in learning/playing activities [32].

As one of the main types of technology-enhanced language learning [7], GBLL was widely reported as significantly useful for language learning [5, 7]. For example, [33] proposed a cognitive complexity-based game. While playing this game, learners received instructional content and took learning tasks. The difficulty of the game varied according to their cognitive capacities. The results showed that the proposed GBLL system significantly improved language learners’ performance in vocabulary tests and reduced their anxiety. Similarly, [34] developed a task-based, augmented reality-enhanced GBLL that situated language learners to develop and apply knowledge in a meaningful three-dimensional context. The results showed that the students who experienced GBLL outperformed those who experienced traditional learning methods in the post-test.

The previous studies explained the effectiveness of GBLL with its positive effects on promoting learner motivation [35]. Drawing on the entertainment value of games [36], GBLL is inherently more exciting and attractive than the conventional learning methods to encourage students to initiate and sustain learning [37]. During the process of GBLL, students tended to engage deeply in educational contents with high concentration, forget all irrelevant issues and time passing, feel enjoyment and a sense of achievement, control over the entire process, and eventually achieve enhanced motivation [27, 30, 34]. Empirical research [13] was undertaken to develop a badge mechanism-enhanced English as a Foreign Language (EFL) game and investigate its effects on 50 primary students. The results showed that the proposed game was significantly effective in enhancing learners’ motivation in language learning.

In addition, the effectiveness of GBLL may be related to its promoting effect of learner self-efficacy [12, 13]. Self-efficacy refers to “beliefs in one’s capabilities to organize and execute the courses of action required to produce given attainments” [38]. It is a significant predictor of language learning efficiency, predominantly affecting learners’ decision-making and problem-solving abilities and their learning persistence in the face of challenges [38, 39]. In GBLL, learners tended to feel a strong sense of control and a clear idea of their learning goals and ways, so they developed a high level of self-efficacy during the process [14, 30, 34]. For example, [14] developed a Kinect technology-enhanced GBLL system through which students could practice their English communication skills in real-life scenarios. The results showed that the students who used the system had significantly higher self-efficacy than those who did not.

To enhance the efficiency of GBLL, researchers suggested increasing learner autonomy. As identified in [28, 34], learners/players who were afforded full autonomy in GBLL tended to immerse into the learning/playing activities with high concentration and great excitement, resulting in high learning efficiency. Additionally, [40] reported that language learners with higher autonomy were more likely to participate in various GBLL-related activities, such as the translation of in-game texts and the communications about the game in online forums, which would further enhance the positive effects of GBLL on language learning.

### 3.2 Concepts of SRLL

Self-regulated learning refers to an educational approach through which learners plan the actions, conditions and other factors that affect their learning [8, 15, 16]. Drawing on the dimension of “locus of control” of autonomous learning, self-regulated learning emphasises the development of learners’ own ability to select and use different learning strategies to control their study with minimal reliance on teachers [41].

Previous researchers have proposed different models and frameworks of self-regulated learning, among which Barry Zimmerman's Cyclical Phases Model was widely cited [1, 41]. The Cyclical Phases Model [15, 16] argues that the process of self-regulated learning consists of three phases: forethought, performance, and self-reflection. In the forethought phase, learners analyse their competencies, determine their needs, set learning goals, and plan their way to reach goals. In the performance phase, learners work on the learning tasks with a high level of concentration, monitor their learning processes, make in-time adjustments to their learning goals and strategies, and seek help when needed. In the self-reflection phase, learners summarise and reflect on the entire learning progress and the used strategies to prepare for future learning.

Besides the phases, the implementation of self-regulated learning involves four categories of strategies, specifically, metacognitive strategies, cognitive strategies, motivational strategies, and behavioural strategies [1, 2]. Metacognitive strategies concern high-order skills interacting with the other three categories that learners use to monitor, regulate, and control the entire learning process [16, 42]. Four metacognitive strategies were frequently investigated in the previous SRLL studies. Specifically, (a) goal setting, that refers to setting goals and sub-goals of learning [15, 43]; (b) planning, that concerns arranging the time, types, and sequence of various learning tasks [4, 16]; (c) self-monitoring, that refers to tracing and recording learning events and results [43]; and (d) self-evaluation, that is evaluating the quality or progress of learning by comparing the learners’ current performance with their learning goals or standard criteria [16, 43]. Cognitive strategies refer to the skills to accomplish learning tasks and goals using cognitive abilities [15, 16]. Example strategies frequently investigated in the previous studies include (a) rehearsal, that is related to memorising learning materials through exercise and repetition [4, 43]; and (b) record reviewing, that is related to recalling learning events and rereading learning materials [4, 15]. Motivational strategies are emotion management skills to initiate and sustain learning [16, 42]. Two motivational strategies were widely investigated in the previous studies, namely, (a) effort and emotion regulation, that is about addressing negative emotions, prompting motivation, enhancing learning awareness, improving learning persistence, and reducing stress in learning [15, 16, 43]; and (b) self-consequence, that concerns accepting or arranging rewards or punishment depending on the learners’ competition or performance in learning tasks [43]. Behavioural strategies encompass the skills to select or create a positive physical and social environment for learning [42]. Peer learning and feedback handling were frequently investigated in the previous studies, which refer to language learners working on academic projects with their peers and responding to their peers’ comments, instructional supports, and encouragements [16, 43]. According to the Cyclical Phases Model, learners practise goal setting and planning in the forethought phase; they practise rehearsal, effort and emotion regulation, self-monitoring, peer-learning and feedback handling self-consequence in the performance phase; they practise record reviewing and self-evaluation in the self-reflection phase.

In the context of language education, the effects of self-regulated learning have been extensively investigated and reported to be overall positive [3, 44, 45]. For example, [46] designed an online course on speaking skills and required students to perform various self-regulated learning strategies, such as planning and self-monitoring. The results showed that the students who frequently employed self-regulated learning strategies in the course tended to outperform those who did not in the post-test. Similarly, [6] required 303 students to experience self-regulated vocabulary learning and complete a post-test and a questionnaire. The results revealed that the learning approach was significantly effective in developing learners’ vocabulary knowledge.

The effectiveness of SRLL may be attributed to the enhanced learner self-efficacy, motivation, and autonomy in this learning approach [10, 11, 47]. Empirical evidence was found in [10], which identified the positive effects of students’ use of self-regulated learning strategies, especially goal setting and planning, on their language learning outcomes. It was explained that by using these strategies, students were more likely to focus on the specific goals and sub-goals of their learning, foresee their whole learning process, and mentally attend to the specific steps required to reach the goals. Those mental activities increased students’ sense of control over their learning and confidence in fulfilling the learning tasks, thereby raising their self-efficacy, motivation, and autonomy in learning.

Moreover, learners’ enhanced self-efficacy may positively affect their outcomes of SRLL [5, 48]. For example, [47] identified positive correlation between students’ practice of SRLL and self-efficacy through questionnaires. They added that students who felt more efficacious tended to devote more effort to learning activities. As a result, students who were more likely to use self-regulated learning strategies tended to be able to optimise their learning actions and factors.

### 3.3 History of the interdisciplinary field between GBLL and SRLL

**Fig 1:**
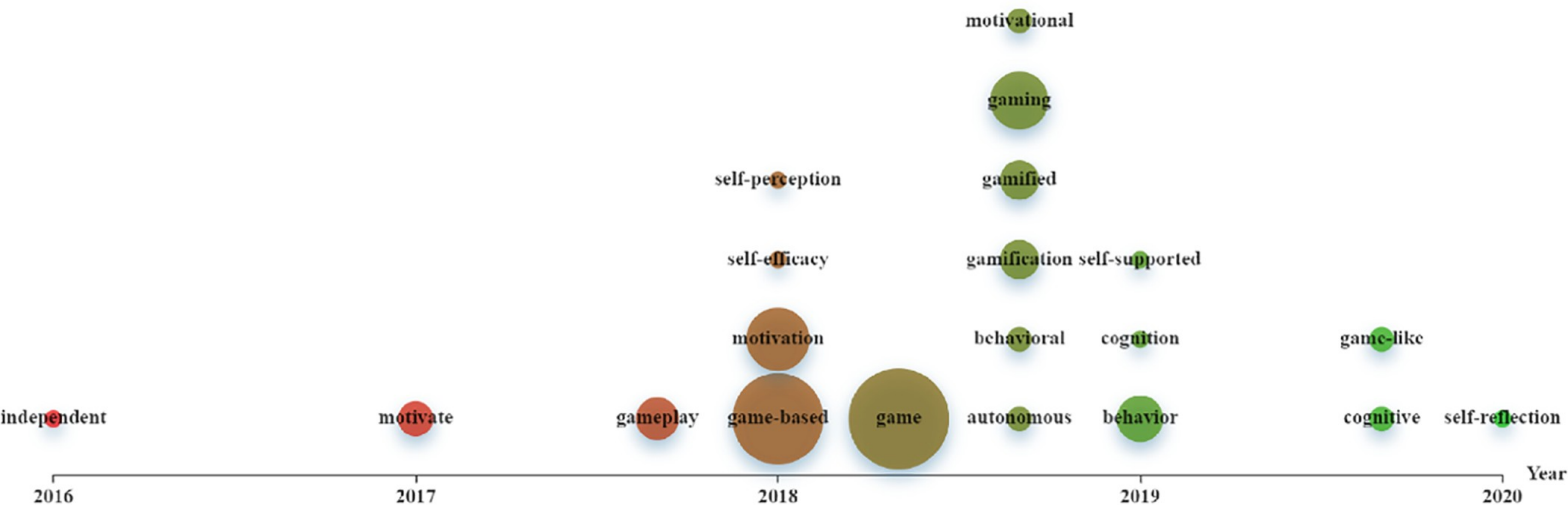
Graphing emergence of the topics of self-regulated learning in game-based language learning studies.

We traced the history of the interdisciplinary field between GBLL and SRLL by investigating when the papers on the interdisciplinary topics were published. Fig 1 illustrates the emergence of the SRLL-related topics in the field of GBLL. It is shown that the topics of self-regulated learning (e.g., *Self-efficacy*, *Self-perception*, *Motivational*, *Behavioural*, *Autonomous*, *Self-supported*, *Cognition*, *Behaviour*) have started being investigated in the papers on GBLL since the period from 2018 to 2019.

**Fig 2:**
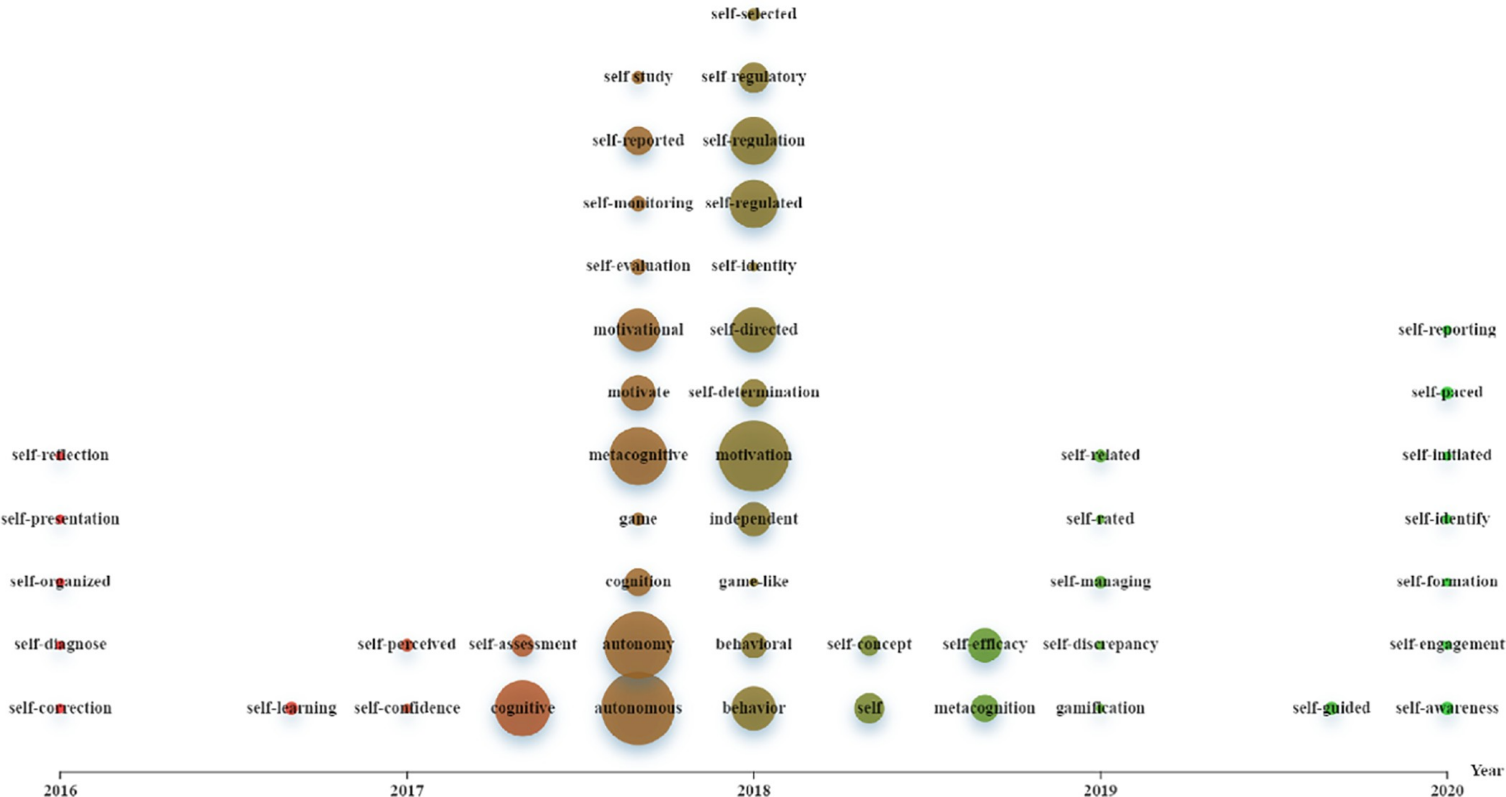
Graphing emergence of the topics of game-based learning in self-regulated language learning studies.

Similarly, Fig 2 illustrates the emergence of GBLL-related topics in the field of SRLL. It shows that the topics of game-based learning (e.g., *Game*, *Game-like*, *Gamification*) have started being investigated since the period from the end of 2017 to 2019.

In sum, the history of the interdisciplinary field between GBLL and SRLL may be traced to the period from 2018 to 2019.

### 3.4 Prominent research topics in the interdisciplinary field between GBLL and SRLL

**Fig 3:**
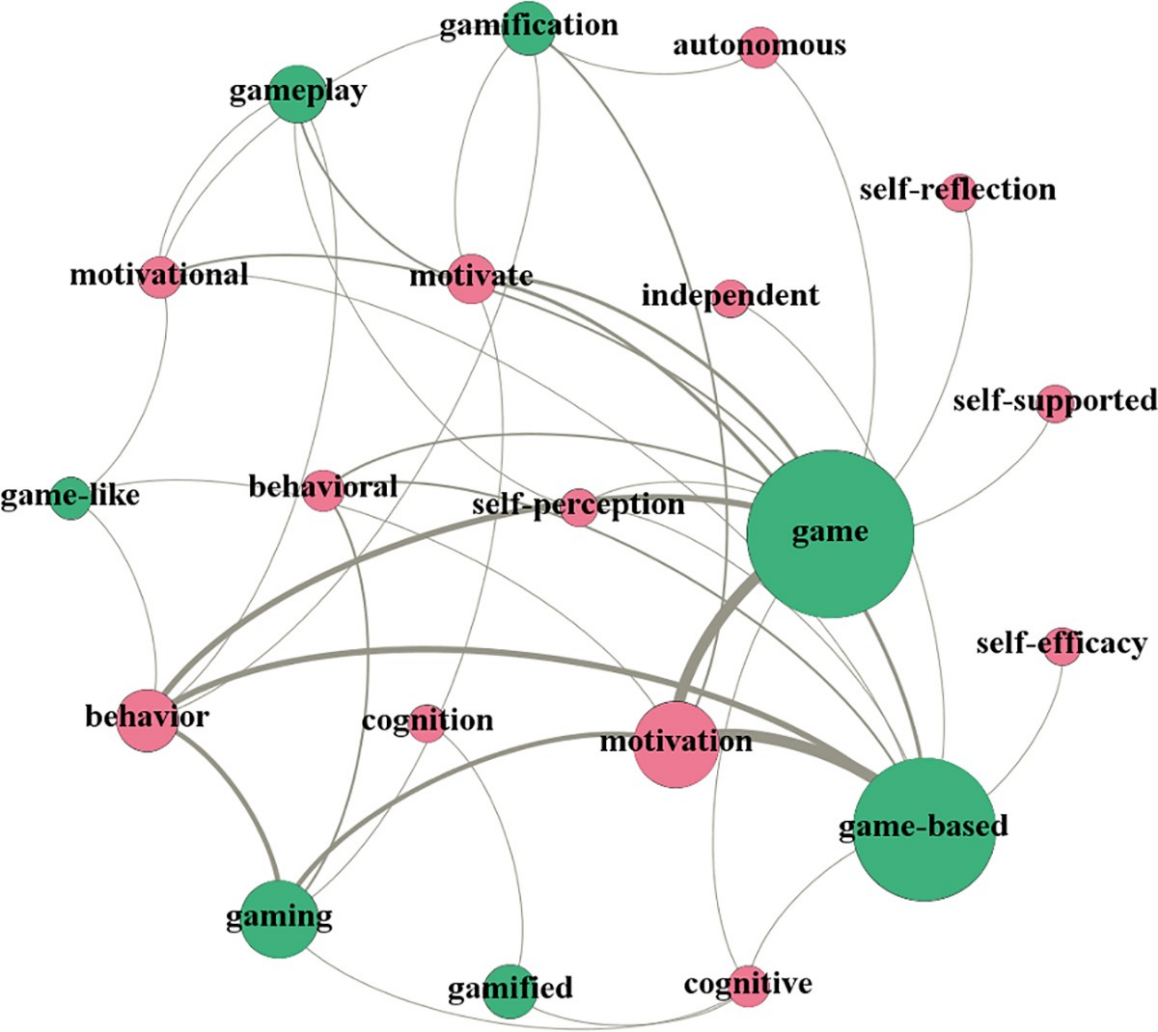
Graphing positive correlations of the topics of self-regulated learning with game-based learning studies.

**Fig 4:**
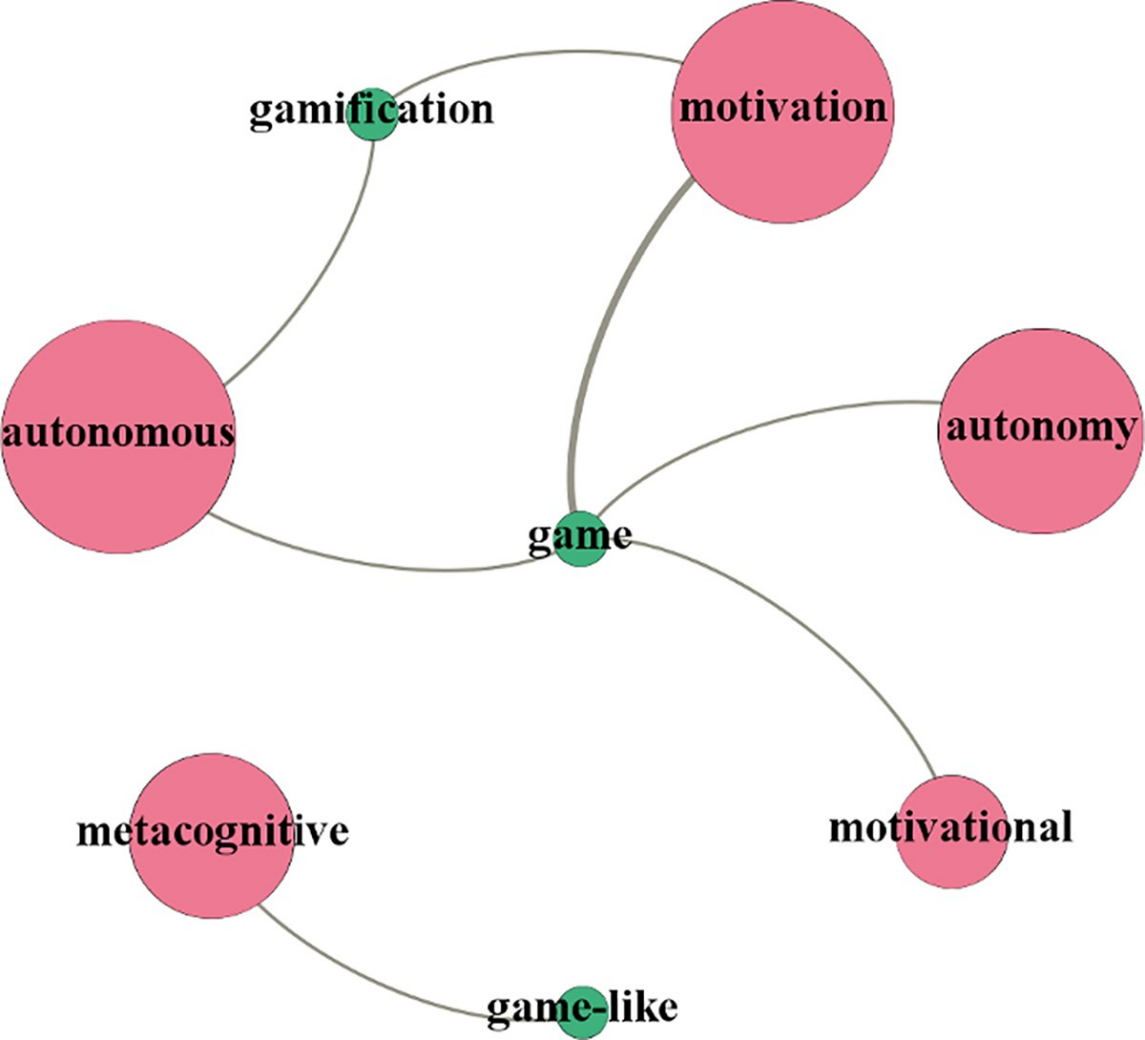
Graphing positive correlations of the topics of game-based learning with self-regulated learning studies.

The study investigated the prominent research topics in the interdisciplinary field between GBLL and SRLL by examining the topical correlation between the topics of these two fields. The topical correlation was analysed from two dimensions: (a) the correlations of the SRLL-related topics with the GBLL studies, as shown in Fig 3, and (b) the correlations of the GBLL-related topics with the SRLL studies, as shown in Fig 4. In the generated diagrams, topics are represented by nodes where green nodes are for GBLL-related topics and red nodes are for SRLL-related topics. The node size reflects the publication counts of the topics relative to the dataset of the corresponding discipline.

Fig 3 reveals three main SRLL-related topics that were discussed in the GBLL studies. One concerns the self-regulated learning strategies that can be performed in GBLL, including topics as *Behavioural*, *Behaviour*, *Motivational*, *Self-reflection*, *Cognitive*, and *Cognition*. The second focus concerns the effects of the combination of GBLL and SRLL on language learning, covering topics as *Motivation*, *Motivate*, *Self-efficacy*, and *Self-perception*. The third focus concerns the SRLL features in GBLL, encompassing the topics as *Autonomous*, *Self-supported*, and *Independent*. Since the sizes of the nodes representing *Motivation* and *Behaviour* are biggest, learner motivation and behaviour as well as their associated strategies were mostly investigated in the GBLL studies involving SRLL.

Fig 4 shows that two main GBLL-related topics were often investigated in the SRLL studies. One topic is about the effects of the combination between GBLL and SRLL, covering topics of *Motivation*, *Autonomy*, and *Autonomous*, which suggests that learner motivation and learning autonomy were mostly investigated in the SRLL studies involving GBLL. The other topic concerns the GBLL features in SRLL, including topics of *Game-like* and *Metacognitive*, which indicates that the game-like features of metacognitive strategies were often investigated in this field.

In sum, this study identified three prominent research topics in the interdisciplinary field between GBLL and SRLL. Specifically, (a) behavioural strategies, motivational strategies, cognitive strategies, and self-reflection as the SRLL strategies that can be performed in GBLL; (b) the effects of the combination of GBLL and SRLL on motivation, self-efficacy, learning autonomy, and self-perception; and (c) game-like, autonomous, self-supported, and independent as the features of the combination of GBLL and SRLL.

## 4. Discussion

Given the above results, RQ4 was addressed by conceptualising GBSRLL and suggesting future research directions.

### 4.1 Concepts of GBSRLL

Based on the in-depth analyses on the two datasets, we can conclude the definition of GBSRLL as the integration of GBLL and SRLL, that is, the language learning approach in which language learners perform self-regulated learning strategies to control their game-playing as the learning process. The process of GBSRLL is game-like, in which language learners engage in learning tasks autonomously and independently by performing various learning strategies, such as behavioural strategies, motivational strategies, cognitive strategies, and self-reflection. GBSRLL can be conducive to learner motivation, self-efficacy, learning autonomy, and self-perception. These results are consistent with [5]’s findings, who developed an app for GBSRLL integrated with virtual reality technology and reported that the app could enable students to engage deeply in learning tasks and develop their self-efficacy with enjoyable learning experiences.

**Fig 5:**
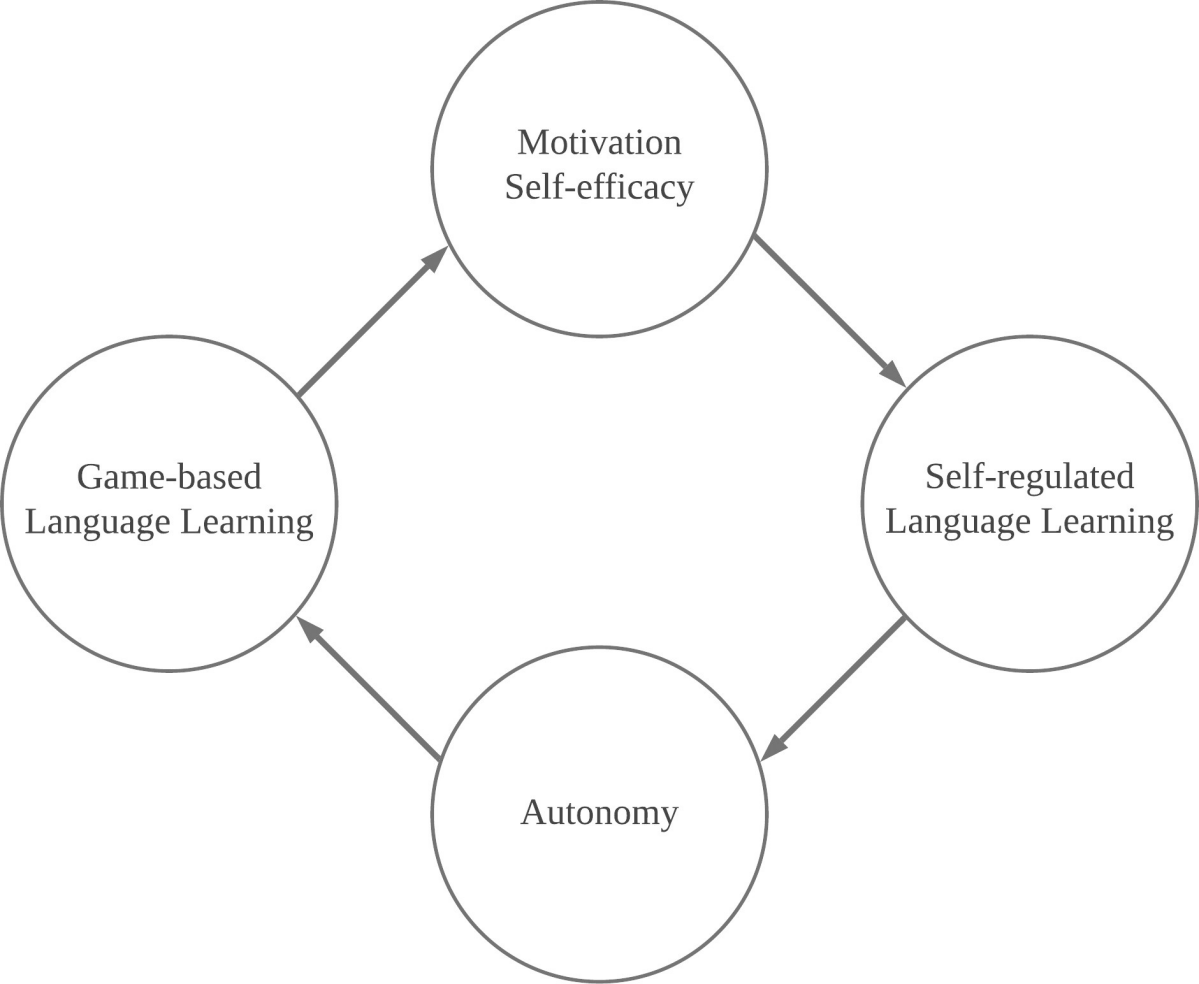
Positive correlations between learners’ motivation, self-efficacy, and autonomy and their implementations of game-based language learning and self-regulated language learning.

Theoretically, GBSRLL can be effective to language learning because of the positive correlations between learners’ motivation, self-efficacy, and autonomy and their implementation of GBLL [12, 13] and SRLL [5, 48], as illustrated in Fig 5. While being engaged in GBSRLL, language learners can achieve a high level of motivation and self-efficacy due to their enjoyment and sense of control in game-based learning [14, 30]. The learners’ raised motivation and self-efficacy would in turn lead to their increased effort for SRLL to control and optimise their learning by proactively performing self-regulated learning strategies [47, 48]. Effective self-regulated learning enables learners to be more self-efficacious about the target language and language learning activities, resulting in satisfying learning outcomes [10, 11]. Additionally, the implementation of SRLL affords the learners with higher levels of autonomy in learning, encouraging them to implement GBLL more efficiently and participate in GBLL-related activities more actively, which may further enhance the effectiveness of GBLL. A cyclic effect is thus established based on the positive correlations between learners’ motivation, self-efficacy, and autonomy and their implementation of GBLL and SRLL. Empirical evidence supporting this effect can be found in [12], which developed a game-based self-regulated language learning app and examined its impacts on 146 students. The results showed a positive correlation between learners’ self-efficacy, self-regulated learning strategies, and motivation in GBSRLL.

Practically speaking, GBSRLL is feasible because game mechanics can support various phases and strategies of self-regulated learning, as shown in Table 1. In the forethought phase, Goals/Rules and Mystery/Fantasy can facilitate language learners to set goals and plan their studies [27, 28]. In the performance phase, learners are provided with opportunities to receive instructional content and rehearse their language skills and knowledge through game playing [5]. Competitions/Contests can help language learners regulate their emotion and effort in learning by guiding them to engage in learning tasks with high concentration, dedicated effort, and great excitement [31]. Interactivity/Feedback in peer-learning and feedback handling can reduce students’ sense of loneliness and sustain their engagement in learning activities for a long time [27], leading to their effort and emotion regulation. Interactivity/Feedback can also cultivate students’ abilities and awareness of self-monitoring under the guidance of NPCs. Rewards/Points can attract learners to practise self-consequence autonomously and devote more effort to learning tasks. In the self-reflection phase, learners can be supported to review their entire learning process by revisiting the game's Mystery/Fantasy. NPCs can remind learners to perform record reviewing through Interactivity/Feedback. Finally, when calculating how many Rewards/Points they have won in various learning tasks, learners can undertake self-evaluation of their previous learning performance.

**Table 1:** Implementation of GBSRLL.

Phases of self-regulated learning	Self-regulated learning strategies	Game mechanics
The forethought phase	Goal setting	Goals/Rules
	Planning	Mystery/Fantasy
The performance phase	Rehearsal	Competitions/Contests
	Effort and emotion regulation	Interactivity/Feedback
		Competitions/Contests
	Self-monitoring	Interactivity/Feedback
	Peer-learning and feedback handling	Interactivity/Feedback
	Self-consequence	Rewards/Points
The self-reflection phase	Record reviewing	Mystery/Fantasy
		Interactivity/Feedback
	Self-evaluation	Rewards/Points

Based on the theoretical framework and the implementation methods, GBSRLL can be a promising and feasible approach to language education that is potentially conducive to learning efficiency.

### 4.2 Future directions of GBSRLL

Despite the great potential of GBSRLL, the research in this interdisciplinary field has remained very limited and it is necessary to call for more research into this language learning approach. For example, most prior studies of GBSRLL focused on its effects on learners’ affective states such as learner motivation, self-efficacy, and learning autonomy (e.g., [5, 12]), as indicated by the results of the social network analysis in this study. More research is needed to investigate the specific effects of GBSRLL on learners’ academic performance in various aspects of language knowledge and skills. Furthermore, the investigation of the effects of GBSRLL should last over a long period of time since GBSRLL may have long-term effects on learners’ lifelong language learning process. The effectiveness of GBSRLL can also be compared with other language learning approaches, including GBLL and SRLL, to gain a more comprehensive understanding of this approach.

GBSRLL is worthy of exploration at the technical level. Tools and technologies are essential for technology-enhanced language learning [7], so the implementation and effectiveness of GBSRLL may be influenced by the tools and technologies used therein. While mobile technology and virtual reality technology have been found supportive for this learning approach (e.g. [5, 12]), other types of tools and technologies that have not yet been thoroughly studied may also be useful. For example, researchers have integrated augmented reality technology into game-based learning and reported its significantly positive effects on improving learning experience and boosting learner motivation [34]. This technology was also reported as effective in enhancing the effectiveness of SRLL by increasing learner attention and reinforcing learning materials [49]. Thus, augmented reality technology may be effective in supporting GBSRLL and can be investigated in future research.

## 5. Conclusion

This study was designed to investigate the concepts of GBSRLL by conducting a combination of theoretical analysis, thematic evolution analysis, and social network analysis on the research articles in the fields of GBLL and SRLL. The results show that GBSRLL is a new interdisciplinary field that has been attracting increasing academic attention since the period from 2018 to 2019. The prominent research topics in this field include (a) self-regulated learning strategies that can be performed in GBLL, (b) the effects of GBSRLL on learners’ affective states, and (c) the features of GBSRLL. As a language learning approach, GBSRLL is a game-like process in which learners take charge of their language learning autonomously and independently by performing various learning strategies, such as behavioural strategies, motivational strategies, cognitive strategies, and self-reflection. The effectiveness of GBSRLL is based on the positive correlations between learners’ motivation, self-efficacy, and autonomy and their implementation of GBLL and SRLL. The practicality of this learning approach is based on the strong supportiveness of game mechanics for various phases and strategies of self-regulated learning. Finally, the results indicate that more contributions can be made to this innovative language learning approach. Two possible directions for future research in this field are suggested: the investigation of the long-term effects of GBSRLL on language learners’ academic performance and the exploration of the supportive tools and technologies for GBSRLL.
